# Graphlets in comparison of Petri net-based models of biological systems

**DOI:** 10.1038/s41598-022-24535-5

**Published:** 2022-12-04

**Authors:** Bartłomiej Szawulak, Piotr Formanowicz

**Affiliations:** Institute of Computing Science, Poznan University of Technology, 60-965 Poznań, Poland

**Keywords:** Computational science, Bioinformatics

## Abstract

Capability to compare biological models is a crucial step needed in an analysis of complex organisms. Petri nets as a popular modelling technique, needs a possibility to determine the degree of structural similarities (e.g., comparison of metabolic or signaling pathways). However, existing comparison methods use matching invariants approach for establishing a degree of similarity, and because of that are vulnerable to the state explosion problem which may appear during calculation of a minimal invariants set. Its occurrence will block usage of existing methods. To find an alternative for this situation, we decided to adapt and tests in a Petri net environment a method based on finding a distribution of graphlets. First, we focused on adapting the original graphlets for notation of bipartite, directed graphs. As a result, 151 new graphlets with 592 orbits were created. The next step focused on evaluating a performance of the popular Graphlet Degree Distribution Agreement (GDDA) metric in the new environment. To do that, we decided to use randomly generated networks that share typical characteristics of biological models represented in Petri nets. Our results confirmed the usefulness of graphlets and GDDA in Petri net comparison and discovered its limitations.

## Introduction

During the last two decades it was more and more evident that living organisms as well as their functional blocks, like organs, tissues, cells, etc., are complex biological systems. It means that for a deep understanding of their nature and functionality it is not sufficient to analyze their building blocks separately (which was a dominating approach in biological sciences). More global view on the relations among elementary biological components is necessary. In other words, living organisms should be seen as complex systems because dense networks of interconnections among their components determine their structure and functionality. So, a systems approach is needed for studying their properties. Such an approach has been for decades applied in technical sciences, so some already developed methods can be adapted to biological research. This led to an emergence of systems biology, i.e., a branch of science, where biological phenomena are analyzed from systems point of view^1^.

A basis for a formal analysis of any system, not only a biological one, is a model expressed in a language of some branch of mathematics. Like in other areas of science and technology, differential equations play an important role also in modelling biological systems. However, in this area they have some serious limitations following, among others, from the fact that usually it is very difficult, or even impossible, to collect all quantitative data characterizing the analyzed system. It follows from the fact that technically it is very hard to measure in a laboratory all parameters describing dozens or hundreds of elementary reactions/processes taken into account in a model. These parameters are necessary in models based on differential equations but are not used in graph-based models since they describe only a structure of the system (which in most cases is crucial for the functionality of biological systems). This is one of the reasons that models based on graph theory are often built and analyzed.

One of the more and more popular approaches to modelling biological systems is based on Petri nets (metabolic pathways, organelles, cells, and drug development^2,3^). Nets of this type are not graphs but they have a structure of a weighted directed bipartite graph^4–6^.

In the analysis of biological systems there happens that there is a need to compare two or more of them. Such a comparison may lead to, e.g., discovering some important, well conserved subprocesses. There is a lot of methods of graph comparison (e.g. graph distance^7^, graph edit distance^8^, maximal common subgraph^9^), but since Petri nets are not graphs, such methods usually cannot be directly applied to comparisons of models expressed in the language of Petri nets theory. Moreover, there is only few methods for comparison of Petri net-based models of biological systems known in the literature^10,11^. Since there is a growing popularity of Petri nets in systems biology, there is also a growing need for such methods.

In this paper we propose a method for Petri nets comparison based on graphlets. They are sets of connected subgraphs and almost two decades ago were proposed for an analysis of protein–protein interaction (PPI) networks^12^. Such networks are undirected graphs describing interactions in a set of analyzed proteins.

The organization of this paper is as follows. In Section Methods basic concepts of Petri nets and graphlets are briefly presented. In “Results and discussion” section graphlets which can be used for Petri nets comparison are proposed. Moreover, in this section results of computational experiments in which Graphlet Degree Distribution Agreement (GDDA) measure was tested in the context of the proposed graphlets, are presented. The last section contains conclusions.

## Methods

### Graphs

Let *V* be a nonempty set and $E \subseteq \{\{v_i,v_j\} : v_i \in V \wedge v_i \in V\}$. The pair (*V*, *E*) is then called a undirected graph where *V* is the set of vertices, or nodes, while *E* is its set of edges. Let *V* be a nonempty set and $A \subseteq V \times V$. The pair (*V*, *A*) is then called a directed graph or digraph, where *V* is the set of vertices, or nodes, while *A* is its set of arcs. Graph $G = (V,E)$ is called a bipartite graph, if $V = V_1 \cup V_2, V_1 \cap V_2 = \emptyset$ and for every edge $\{x,y\} \in E$, there is $x\in V_1 \wedge y \in V_2$^13^.

### Petri nets

As has been mentioned in the previous section, Petri nets are one of mathematical formalisms which can be used to model and analyze biological systems. They have a structure of a biparted, directed graph with weighted arcs. Formally Petri net can be defined as a 5-tuple $Q = (T,P,F,W,M_0)$, where:

- $P=\{p_1,p_2,...,p_n\}$ *and* $T=\{t_1,t_2,...,t_r\}$ *are finite, disjoint sets of places and transitions, respectively; *
- $F \subseteq (P \times T) \cup (T \times P)$ *is a finite set of arcs;*
- $W : F \rightarrow {\mathbb {Z}}^{+}$ *is a weight function;*
- $M_0 : P \rightarrow {\mathbb {N}}$ *is a function assigning number of tokens to each place *—called net state function^4^.

They are composed of vertices of two kinds, i.e., places and transitions, which can be connected by arcs. Places correspond to passive components of a modelled system, while transitions are counterparts of its active components (i.e., some elementary processes). Arcs describe casual relationships between components of these two types, can join only vertices of different types. Places, transitions and arcs constitute a structure of a Petri net, which is a directed bipartite graph. In fact, this graph is weighted, because arcs are labeled by positive integers called weights. Tokens reside in places and flow through a net via transitions. This flow is governed by the transition firing rule. According to it a transition is active if in every of its pre-places, i.e., these ones which directly precede it, a number of tokens is equal to at least a weight of an arc connecting the place with the transition. An active transition can be fired, what means that tokens flow from its pre-places to its post-place, i.e., those ones which directly succeed the transition. The numbers of flowing tokens are equal to the weights of the respective arcs. The flow of tokens correspond to a flow of substances, signals, information, etc. in the modelled system and it brings a kind of dynamics to Petri nets, which is a great advantage of these nets in comparison to graphs in the context of systems modelling. Unfortunately because of computational limitations those flows are not always available to calculate in large networks.

**Figure 1:**
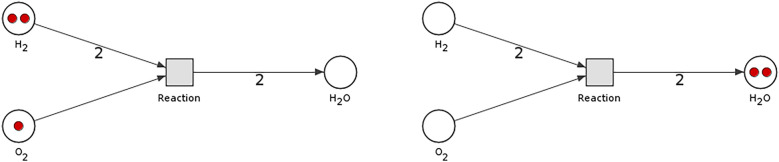
This example of simple Petri net-based model, shows a binding reaction of two hydrogen molecules and one oxygen molecule into one molecule of water (H$_2$O). The left image shows state before binding and the right one after. Red tokens were consumed from a proper places and new one corresponding to a new molecule has been created.

Petri nets have an intuitive graphical representation, where places are depicted as circles, transitions as rectangles or bars, arcs as arrows, and tokens as dots or numbers inside places^4–6^—example on Fig. 1.

The possible flows of tokens are determined by the structure of a Petri net. So, a basis of Petri nets comparison is a comparison of their structures, i.e., the weighted directed bipartite graphs.

**Figure 2:**
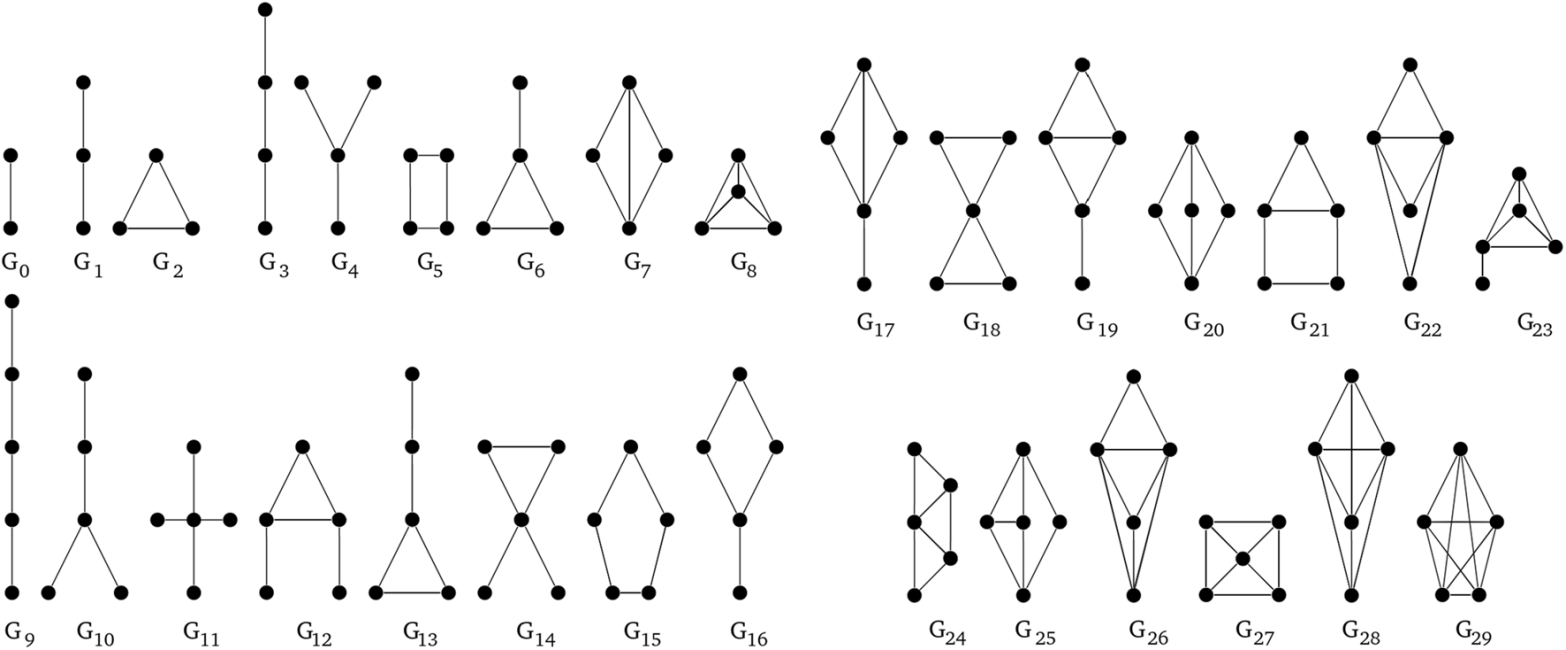
All undirected graphlets build on 2–5 vertices.

### Graphlets

Few of methods based on graphlets, has been used for comparison of graph models of biological phenomena, however, it has not been used for models expressed in the language of Petri nets theory. Undirected graphlets (for short graphlets) were proposed in Ref.^12^ in the context of analysis of PPI networks. The general idea of such an analysis was based on a comparison of frequencies of appearance of some subgraphs in an analyzed PPI network with corresponding frequencies in random graphs (some of related research focus on finding induced subgraphs using graphlets, which is not the topic of this paper). The subgraphs whose frequencies were compared in this approach are connected non-isomorphic graphs on *n* vertices, where n is usually in the range [2, 5]. These graphs are called graphlets^12^. For $n\in [2,5]$ there are 30 graphlets (see Fig. 2). The basic measure, called *relative graphlet frequency distance*, used for comparison of two graphs *G* and *H* based on graphlet frequencies is defined in the following way^12^:

1 $$\begin{aligned} D(G,H)=\sum _{i=0}^{{l}}\left| F_i(G)-F_i(H)\right| , \end{aligned}$$

where $F_i(G)=-\log \frac{N_i(G)}{T(G)}$, $N_i(G)$ is the number of graphlets of *i*-th type (cf. Fig. 2) in graph *G* and $T(G)=\sum _{i=0}^{{l}}N_i(G)$ is the total number of graphlets in graph *G* and *l* is the maximal index of a graphlet. the maximal index of an orbit

**Figure 3:**
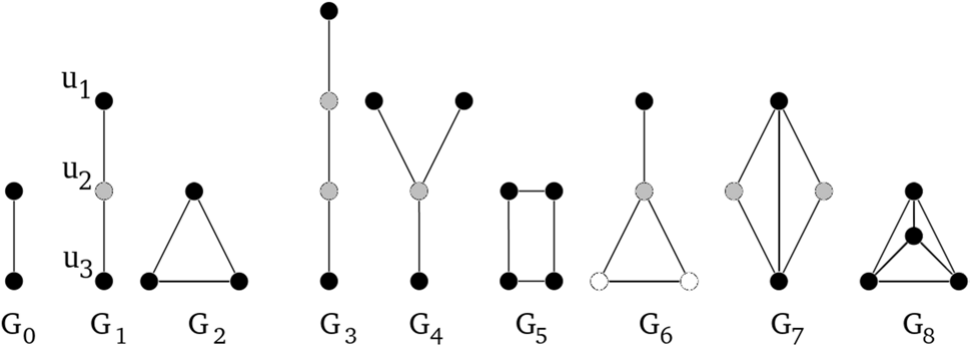
Undirected graphlets with orbits shown in different colors.

As this measure is not precise enough for a significant number of comparison cases (it is easy to construct networks with exactly the same degree distribution whose structure and function differ substantially^14^), another one based on the concept of orbit, was proposed^14^. The idea of orbit follows from the notion of degree distribution. A degree of vertex *v* is the number of edges incident to this vertex. But an edge is the smallest graphlet, i.e., the only graphlet containing two vertices. This simple observation leads to an extension of the notion of degree distribution since it may be considered a number of graphlets built on greater number of vertices which are incident with vertex *v*. However, it is important to distinguish various cases of such an incidency, i.e., which vertex of a given graphlet is incident with vertex *v*. The idea can be easily illustrated on the example of graphlet $G_1$ (see Fig. 3). From topological point of view there is no difference whether vertex $u_1$ is adjacent to vertex *v* or vertex $u_3$ is adjacent to *v*, but these two cases differ from the case, where vertex $u_2$ is adjacent to vertex *v*. So, a set of vertices of a given graphlet can be divided into subsets containing vertices which are equivalent in the already described sense. These subsets are orbits. More formally, an orbit can be defined using the notion of an automorphism—a special case of an isomorphism. Given two graphs $H_1=(V_1,E_1)$ and $H_2=(V_2,E_2)$ an isomorphism is function $f:V_1\rightarrow V_2$, which is a bijection such that for every two vertices $v_1,v_2\in V_1$ edge $\{v_1,v_2\}\in E_1$ if and only if edge $\{f(v_1),f(v_2)\}\in E_2$. An isomorphism of a graph to itself is an automorphism. A set of all automorphisms of given graph $G=(V,E)$ (with an operation of superposition) forms a group called an automorphism group of graph *G*, which is usually denoted by *Aut*(*G*). For vertex $v\in V$ an automorphism orbit or simply orbit of this vertex is set $Orb(v)=\{u\in V: u=f(v), f\in Aut(G)\}$^14^.

Vertices $u_1$ and $u_3$ in graph $G_1$ belong to one orbit, while vertex $u_2$ belongs to another orbit (and these are the only orbits of this graph).

So, now we can measure how many vertices in a given graph are incident with graphlets $G_i$, $i=0,1,\ldots ,l$, where *l* is the greatest index of a graphlet in the considered set of graphlets. We should distinguish the cases of incidency with various orbits in graphlet $G_i$. In other words, we can measure how many vertices are incident with orbits $O_j$, $j=0,1,\ldots ,m$, where *m* is the greatest index of an orbit. For a given automorphism orbit *j*, $d^j_G(k)$ is the sample distribution of the number of nodes in *G* touching the appropriate graphlet *k*-times^14^. However, in this way we obtain a large collection of numbers (degree distributions) characterizing the analyzed graph. They can be arranged in the following matrix^15^:

2 $$\begin{aligned} D_G = \begin{bmatrix} d^0_G(1) &{} d^0_G(2) &{} \dots &{} d^0_G({\alpha})\\ d^1_G(1) &{} d^1_G(2) &{} \dots &{} d^1_G({\alpha})\\ \vdots &{} \vdots &{} \ddots &{} \vdots \\ d^m_G(1) &{} d^m_G(2) &{} \dots &{} d^m_G({\alpha}) \end{bmatrix}. \end{aligned}$$

It should be noticed that in practice, where finite graphs are considered, the matrix is finite, since it is upper bounded by the number of vertices of the analyzed graph (α is the maximal possible number of orbit occurrences in one vertex).

It would be better to have one number, instead of such a matrix, which could be used in comparisons of graphs. A measure of this type has been proposed and called a GDD agreement (GDDA)^14^.

First, $d_G^j(k)$ is scaled:

3 $$\begin{aligned} S_G^j(k)=\frac{d_G^j(k)}{k}, \end{aligned}$$

and then a normalized *j*-th distribution is calculated:

4 $$\begin{aligned} T_G^j(k)= & {} \sum _{k=1}^\alpha S_G^j(k), \end{aligned}$$

5 $$\begin{aligned} N_G^j(k)= & {} \frac{S_G^j(k)} {{T}_G^j(k)}. \end{aligned}$$

Next, for graphs *G* and *H* a *j*-th distance based on their normalized distributions is defined:

6 $$\begin{aligned} D^j(G,H)=\frac{1}{\sqrt{2}}\left( \sum _{k=1}^\alpha \left[ N_G^j(k)-N_H^j(k)\right] ^2\right) ^\frac{1}{2}. \end{aligned}$$

A value of this distance is in the range [0, 1]. When the value is equal to 0, it means that the two graphs have identical distributions, and the greater the value is, the greater differences between the graphs are.

It is also convenient to consider a *j*-th agreement between the two graphs:

7 $$\begin{aligned} A^j(G,H)=1-D^j(G,H). \end{aligned}$$

And on this basis an agreement between graphs *G* and *H* can be defined in the following way:

8 $$\begin{aligned} A(G,H)=\frac{1}{m+1}\sum _{j=0}^{m} A^j(G,H). \end{aligned}$$

The idea of graphlets can be extended to the case of directed graphs. They are called digraphlets. It is worth considering since Petri nets have a structure of a directed bipartite graph. (When graphlets up to size 5 are used, then $l=29$ and $m=72$.)

There are 39 directed graphlets for $n\in \{3,4\}$ and one directed graphlet on two nodes (see Fig. 5). In these graphlets there are 129 orbits^16^.

In this case the directed GDDA (DGDDA) can be defined analogously to the undirected case^16^:

9 $$\begin{aligned} A_d(G,H)=\frac{1}{d+1}\sum _{j=0}^{d} A_d^j(G,H), \end{aligned}$$

where *d* is the maximal index of an orbit in a directed graphlet and agreements $A_d^j(G,H), j=0,1,\ldots ,128$ are calculated for such orbits.

## Results and discussion

### Graphlets for Petri nets

A problem of finding all graphlets in compared models might be the largest obstacle for application of graphlet based methods, especially in large networks. To resolve this problem a numerous heuristics and exact algorithms has been proposed. This paper focuses on determining if graphlet based measures are useful for comparison of Petri net-based models of biological systems. Since we concentrate on properties of graphlets and not on algorithms, a simple greedy method is used for this research.

Petri nets were proposed for modelling concurrent, parallel processes and initially used as representation for automata theory problems^4^. For many years the main area of their applications was computer science or more generally, technical systems, but recently they are more and more often used for modelling and analysis of complex biological systems^2,17^.

**Figure 4:**
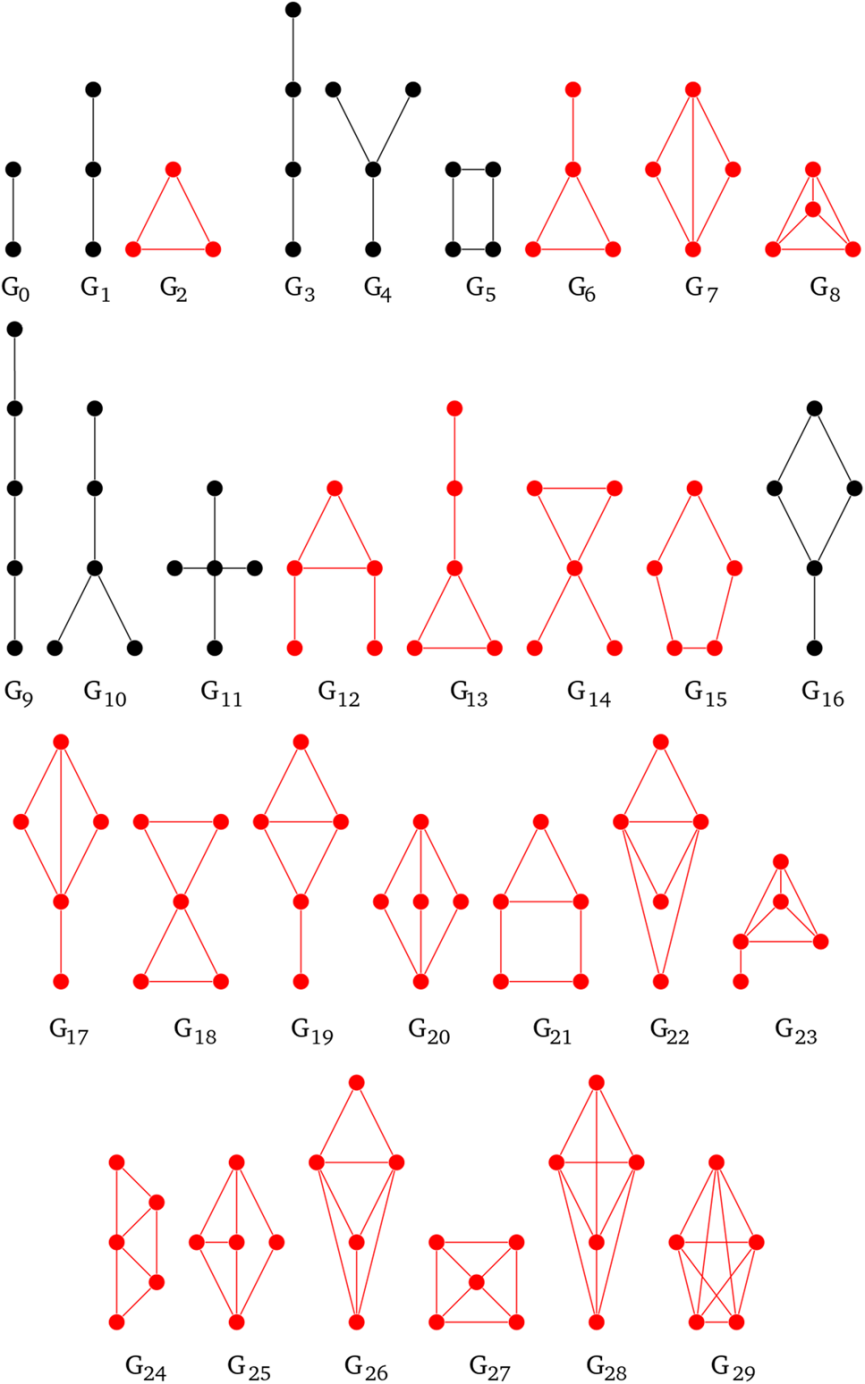
All undirected graphlets build on 2, 3, 4 and 5 vertices. The ones in black color after modifications could occur in Petri nets, while the red ones do not fulfill bipartite property.

**Figure 5:**
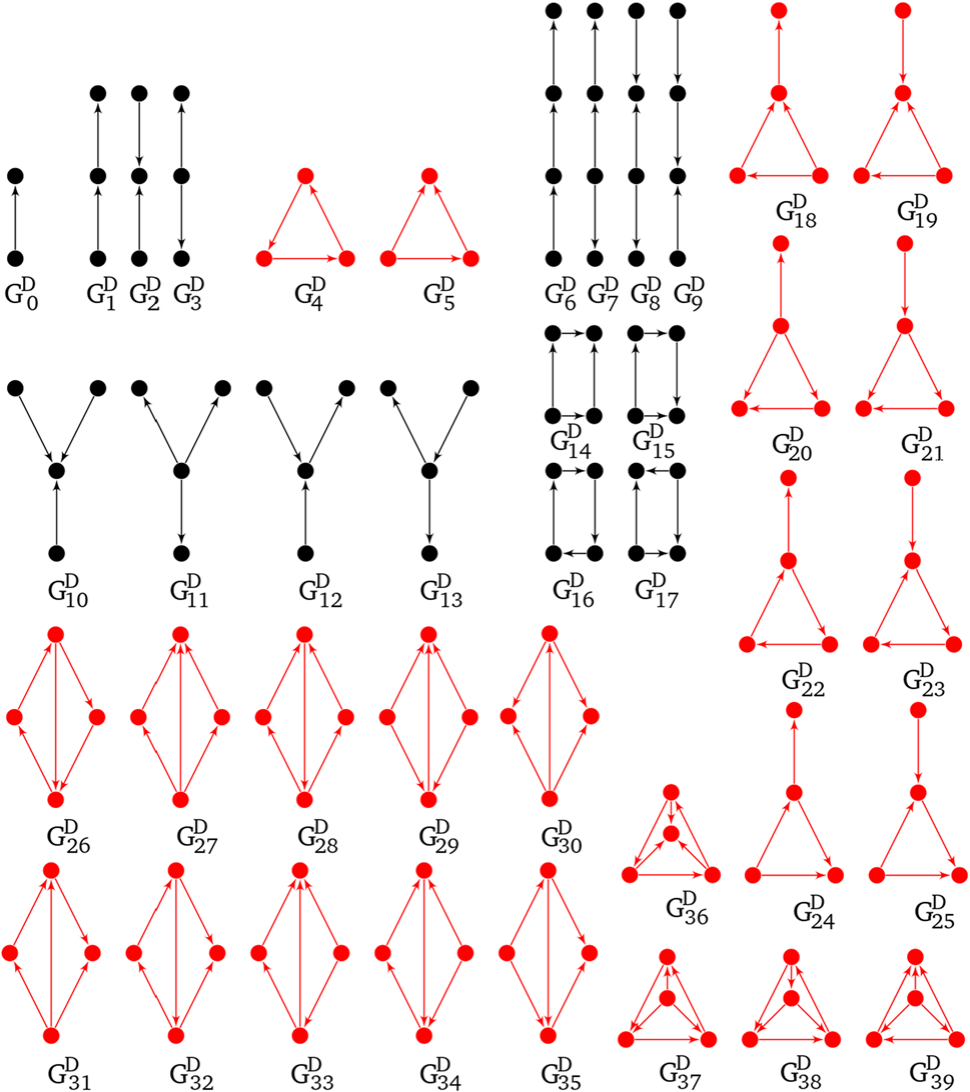
All directed graphlets build on 2, 3 and 4 vertices. The ones in black color can occur in Petri nets.

Petri net-based models, that can be found in practical applications (e.g., industrial), usually do not have size comparable to average PPI nets (they are usually smaller). However, in a biological context models based on them can have hundreds of nodes or more.

Despite their popularity, there are only two methods for comparison of Petri net-based models of biological systems^10,11^ (both use matching of invariants or structures based on invariants, and because of that are potentially vulnerable to the state explosion problem^18^). The reason of this situation can be the fact that it is not an easy task to adapt exact methods of graph comparison to Petri nets. Comparison algorithms for digraphs have higher complexity than their counterparts for undirected graphs. On the other hand, for bipartite graphs there are efficient dedicated methods. For Petri nets both aspects need to be taken under consideration. Because of that, the possibility of exploiting a graphlet-based approach is interesting.

It is not difficult to realize that since Petri nets have a structure of a directed bipartite graph, directed graphlets (which will be called digraphlets) are natural candidates for a good starting point for a construction of a Petri nets comparison method^15,16^.

In 2017 two extensions to directed graphlets were proposed^15,16^ and have been used as a base for graphlets dedicated to Petri nets. Additional modifications like distinction between vertex types (Fig. 7) were needed. Not every digraphlet can occur in a Petri net because of its bipartite structure, i.e., digraphlets with cycles of odd length do not fit this structure and have not participated in further modifications (Figs. 4,  5). It should be mentioned that graphlets with parallel arcs were not considered in this paper.

In result digraphlets $G^D_0$–$G^D_3$, $G^D_6$–$G^D_{17}$ and undirected graphlets $G_9$, $G_{10}$, $G_{11}$, $G_{16}$, $G_{20}$ have acceptable structure (see Figs. 4, 5).

**Figure 6:**
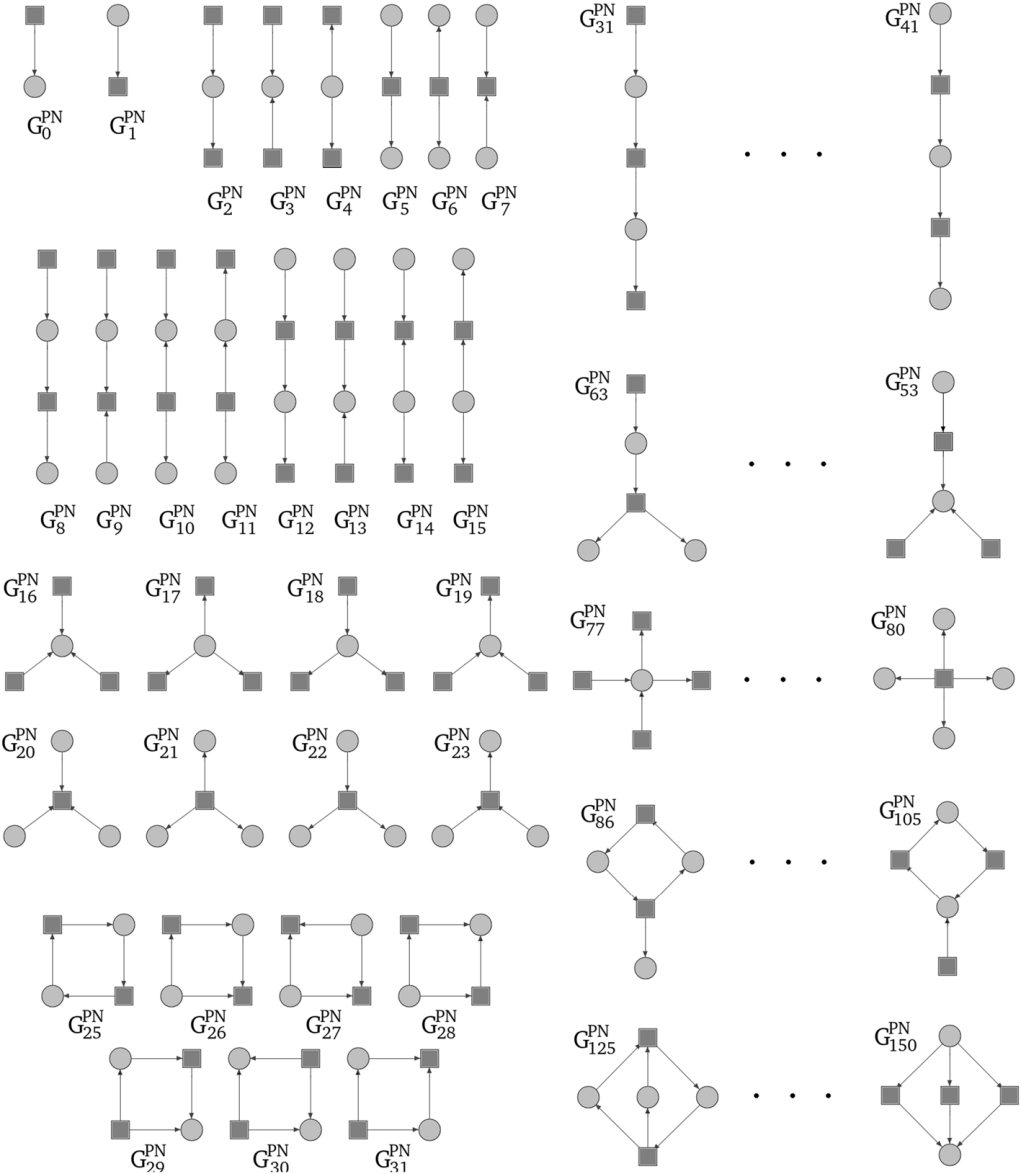
Graphlets for Petri net up to 5-node size.

**Figure 7:**
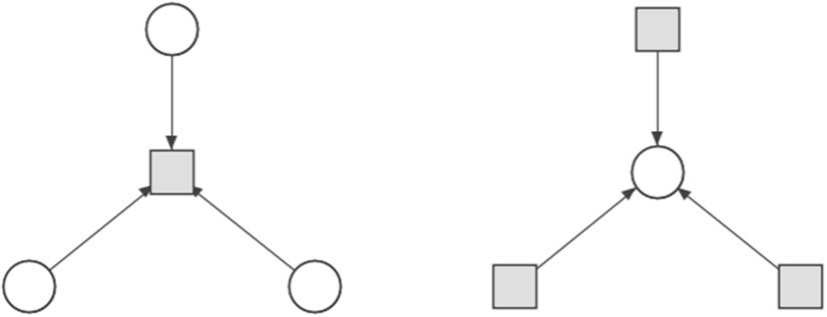
Two variants of digraphlet $G^D_{10}$.

Since in Petri nets there are two types of vertices (i.e., places and transitions) each digraphlet suitable for Petri nets comparison can exists in two variants that have the same structure but types of vertices change to opposite (i.e., a place in one variant will be changed to a transition in second one and vice versa), what is shown in Fig. 7. For example, the simplest digraphlet $G_0^D$ can have two variants, i.e., the one, where the transition has an incoming arc, and the one, where the transition has an outgoing arc. An exception are digraphlets based on graphlet $G_{{16}}$ which has a form of directed cycle. Graphlets adapted to Petri nets environment will be called *pn-graphlets*. They are shown on Fig. 6.

**Table 1:** Number of pn-graphlets and orbits.

Graphlet size	Graphlets		Orbits	
	Count	$\Sigma$	Count	$\Sigma$
2-Node	2	2	4	4
3-Node	6	8	14	18
4-Node	23	31	72	90
5-Node	120	151	502	592

Figure 7 and Table 1 shows for pn-graphlets of sizes in the range [2, 5] the number of all possible structures of a given size. Columns denoted by $\Sigma$ show the number of pn-graphlets or orbits up to the specified size.

GDDA for Petri nets (PN-GDDA) is based on the proposed pn-graphlets:

10 $$\begin{aligned} A_{pn}(G,H)=\frac{1}{p+1}\sum _{j=0}^{p} A_{pn}^j(G,H), \end{aligned}$$

where *p* is the maximal index of an orbit in a pn-graphlet and agreements $A_{pn}^j(G,H)$ are calculated for orbits in such pn-graphlets. Since for pn-graphlets build on 2, 3, 4 or 5 vertices there is 592 orbits, $p=591$ if such pn-graphlets are considered.

**Figure 8:**
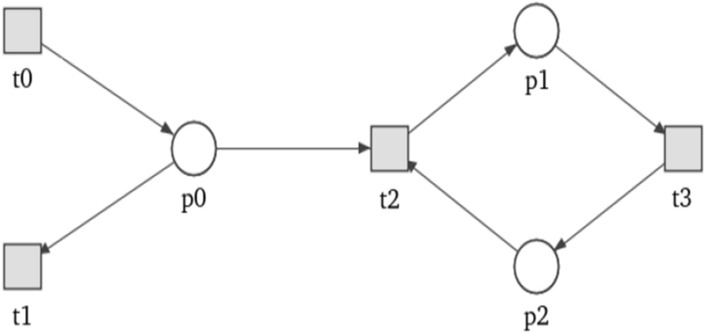
Net used for orbit distribution shown in Table 2.

**Figure 9:**
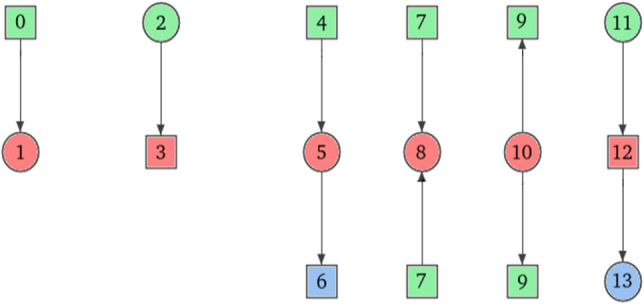
First six pn-graphlets with orbits counted in Table 2.

Figure 8 contains an example of a simple Petri net. It is a 7-vertex connected net with one cycle, one source transition ($t_0$) and one sink transition ($t_1$) (vertices that have only incoming or outgoing arc, respectively). For this network Table 2 contains distribution of orbits from the first 6 pn-graphlets ($G^{PN}_0{-}G^{PN}_5$—see Fig. 9). Columns in this table represents orbits while rows correspond to vertices. A value in a given cell is a number of corresponding orbit occurrences in a given vertex. Orbits in pn-graphlets are strictly connected to specific type of a vertex (c.f. Fig. 9). For example, orbit 0 can occur only in transitions and because of that its value for occurrence in places will always be 0 (situation highlighted by gray color in Table 2).

**Table 2:** Numbers of occurences of each orbit in a specific vertex in the exemplary Petri net shown in Fig. 8.

Orbits	0	1	2	3	4	5	6	7	8	9	10	11	12	13
p0	0	1	2	0	0	2	0	0	0	0	1	1	0	0
p1	0	1	1	0	0	1	0	0	0	0	0	1	0	2
p2	0	1	1	0	0	1	0	0	0	0	0	1	0	1
t0	1	0	0	0	2	0	0	0	0	0	0	0	0	0
t1	0	0	0	1	0	0	1	0	0	1	0	0	0	0
t2	1	0	0	2	1	0	2	0	0	1	0	0	2	0
t3	1	0	0	1	1	0	1	0	0	0	0	0	1	0

### Computational experiments

The earlier described GDDA is a frequently used metric for graphlet-based comparison of networks^14^. It represents an interesting approach to graph comparison, which is efficient in comparing large networks, where more standard measures based on isomorphism (e.g., graph distance, maximal common subgraph, graph edit distance) are not. Complexity of graph isomorphism is still open and comparison methods based on isomorphism are limited to relatively small graphs (e.g., finding maximal common subgraph is an NP-complete problem). Algorithms for finding all (undirected and directed) graphlets have time complexity of $O (nd^{k-1})$^16^, while finding all orbits has complexity $O (ed^{k-3})$^19^ (where *d* is a maximum vertex degree, *n* number of vertices, *e* number of edges), and represent an alternative that can be used in practical applications.

Pn-graphlets are modifications of the basic idea of graphlets that might change some known characteristics of the original graphlets. This possibility pressed us to perform tests that could allow to evaluate performance of comparison methods based on the proposed pn-graphlets and check an impact of this modifications on GDDA value in comparison of Petri net-based models of biological systems.

Such models share a number of common structural characteristics. Defining them, allow to create random networks that fulfils these characteristics, hence could be used for testing GDDA in a biological context. Using such networks gives possibility to test the method on a large number of cases and check its reactions to specific characteristics, i.e., a high ratio of places to transitions numbers.

To simulate biological models random networks should follow a list of restrictions:

1. Each of the nets contains both source and sink transitions or non of them. Transitions of those types represent an interface of the modelled system with its environment
2. A ratio of the number of arcs to the number of vertices is equal to  1.3 (an average ratio). This value is based on Petri net-based models of biological systems available in the literature.
3. They do not contain read nor inhibitor arcs (a read arc correspond to a pair of bi-directed arcs, while an inhibitor arc can be used to prevent transitions from fire in specific net states). While very useful in representation of biological processes, both types of arcs are not compatible with incidence matrix representation, which is a base for many analytical methods. Because of that they are often avoided in biological models.
4. Arc weight can be greater than 1. Weight greater than one can represent approximate quantitative relationships in the modelled biological system.

In models of biological systems, the number of transitions is usually slightly grater than the number of places. However, in order to check pn-graphlet behaviour also in less typical nets, in the tests randomly generated Petri nets with greater differences between the numbers of places and transitions were also used. Because small nets are more vulnerable to differences, nets with size from 20 to 100 vertices, incremented by 5, were used in the experiment. For each pair (*n*, *m*), where $n,m \in [10,50]$ are the numbers of places and transitions, respectively, 100 Petri nets were generated.

#### PN-GDDA and pn-graphlets size

The number of Petri net graphlets is smaller than the number of their classical directed counterparts, which allow for usage of 5-node pn-graphets in all of the performed tests. One of the interesting questions is how a comparison precision changes with the number of pn-graphlets used? To answer this question, a value of PN-GDDA was calculated for Petri net graphlets of sizes 2, 3, 4 and 5.

**Figure 10:**
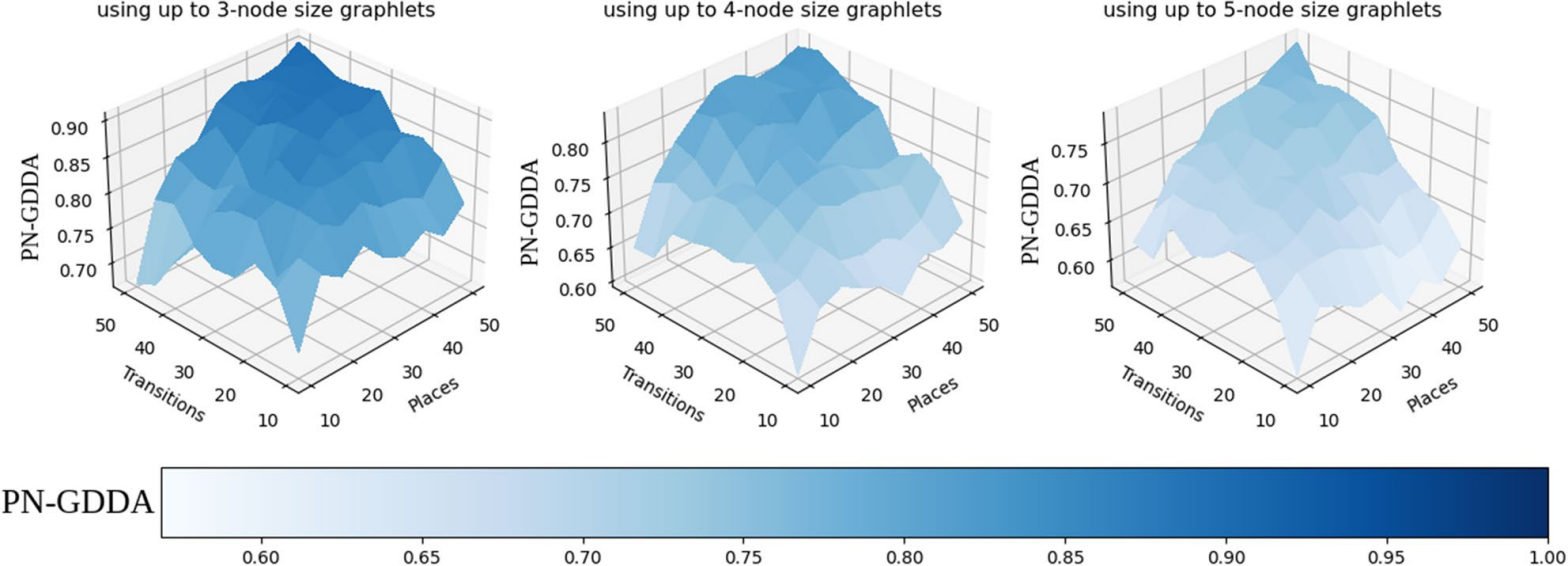
Three diagrams representing results of test determining average values of GDDA (100 nets per case) when pn-graphlets of different sizes are used. Data used for diagrams can be found in Tables S1–S3 from supplementary material. (All diagrams have been created using Seaborn library—v0.11.2—https://seaborn.pydata.org)^20^.

On the three diagrams presented in Fig. 10 there can be observed a reduction of an average similarity value depending on the number of pn-graphlets used to calculate PN-GDDA. Regardless of the size of used pn-graphlets it has been observed that an average similarity rises with the size of the tested networks. As a result of this characteristic, the same value of PN-GDDA metric for net with size 20 and net with size 100 will represent different scale of similarities. This information needs to be considered in interpretation of the comparison results.

**Figure 11:**
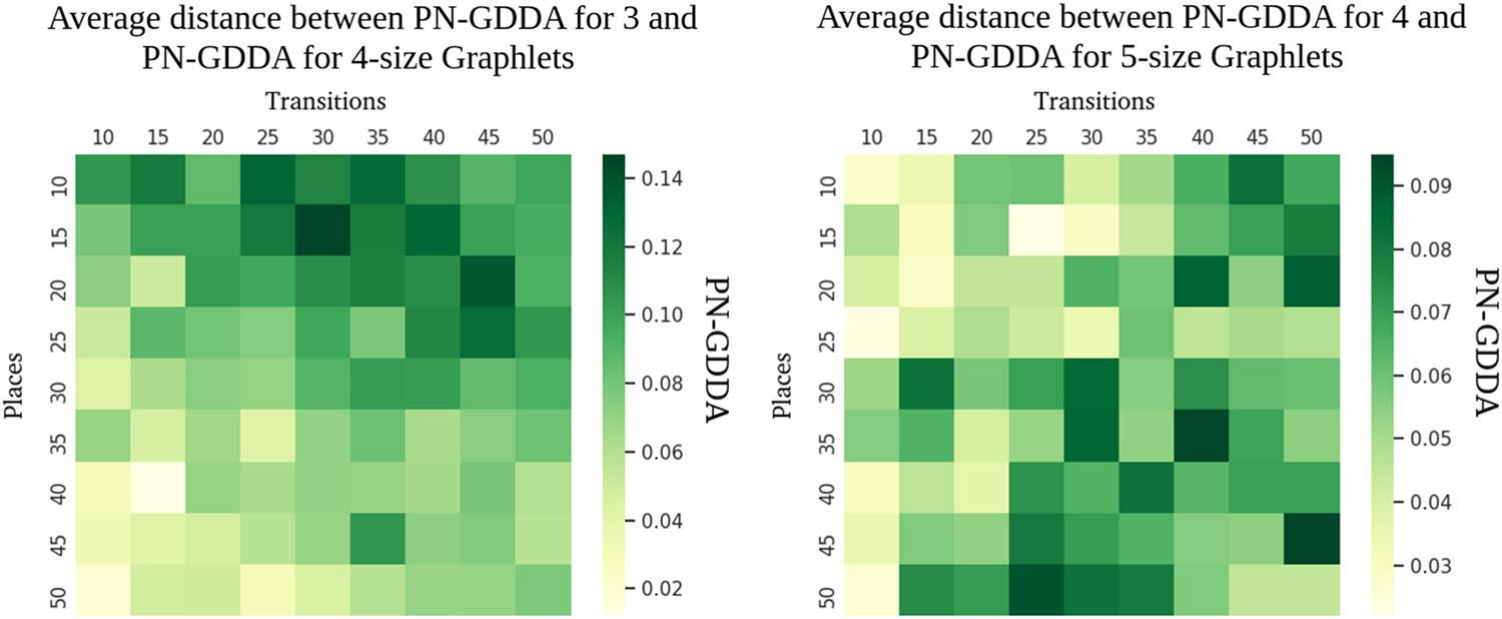
Average similarity level decrese after switching between 3-node and 4-node pn-graphlets (left heatmap), and between 4-node to 5-node pn-graphets (right heatmap)^20^.

For better representation of local changes of PN-GDDA value in response to an increase of a pn-graphlet size two heatmaps are presented in Fig. 11. For increase of the size of pn-graphlets from 3 to 4 nodes an average reduction of GDDA value of 0.081 is observed (from 0.012 to 0.147). The highest decrease of PN-GDDA value was observed for nets with higher number of transitions than places. An increase of pn-graphlets size from 4-node pn-graphlets to 5-node ones results in lower decrease of GDDA value (range from 0.022 to 0.095 with average 0.058). The highest changes are visible for nets with size greater than 50 nodes.

The acquired results confirm that a usage of the largest possible set of pn-graphlets will increase a difference detection levels, which is what could be expected. At the same time it has been observed, that the scale of precision improvement is related to structural characteristic of compared models (e.g., a ratio between the numbers of transitions and places).

#### PN-GDDA and ADT based differences

An interesting question is how PN-GDDA metric reacts to differences, that represent a biologically important substructure? According to Ref.^21^ the ADT (Abstract Dependent Transitions) set is the smallest meaningful element that can be biologically interpreted. Such structure is build around transitions from that set. They correspond to structures based on Maximal Common Transitions sets (MCT sets), which are often used in an analysis of Petri net-based models of biological systems. They allow for a decomposition of a model into a set of subprocesses. A connected variant of ADT is called conADT and they can be members of various graph classes like trees or cycles. We are interested in changes of GDDA in reaction to differences between two compared nets, where difference has a form of ADT-based subgraphs.

In Table 3 a distribution of pn-graphlets in the tested conADT subnets has been presented. It shows that a structural characteristic of subnets can be detected from it, e.g., a high similarity between subnets B,C and smaller with A. In Table 4 the results of a comparison using PN-GDDA for the mentioned previously subnets have been shown. The results also confirm the observations made on the basis of Table 3. While changes in PN-GDDA value are relatively small, it can be observed that a difference in size between the compared pairs can have a significant impact, e.g., PN-GDDA between subnets A–B and A–C is smaller that between A-F. This is caused by a higher number of nodes in subnets B and C, which implies a larger number of pn-graphlets, different numbers of orbits associated with additional nodes and because of that a lower value of PN-GDDA. This should be especially visible on small subnets, where each additional node with an arc creates a number of additional pn-graphlets.

**Table 3:** Distribution of pn-graphlets in structures A–F from Fig. 13.

	A	B	C	D	E	F
Graphlet-0	4	8	8	5	4	3
Graphlet-1	4	8	8	5	4	3
Graphlet-2	4	8	8	5	4	3
Graphlet-5	0	4	4	5	6	4
Graphlet-6	0	0	0	0	1	1
Graphlet-7	6	6	6	0	1	1
Graphlet-13	4	4	4	0	0	0
Graphlet-14	0	0	0	0	2	1
Graphlet-15	0	0	0	0	2	1
Graphlet-16	0	4	4	5	2	0
Graphlet-17	0	0	0	0	2	0
Graphlet-20	12	12	12	0	2	0
Graphlet-21	0	4	4	5	2	0
Graphlet-24	0	0	0	0	2	2
Graphlet-30	0	0	0	0	0	1
Graphlet-31	0	4	4	5	2	0
Graphlet-34	0	0	0	0	1	0
Graphlet-37	6	6	6	0	1	0
Graphlet-41	0	0	3	5	0	0
Graphlet-48	0	12	3	0	0	0
Graphlet-64	0	0	0	0	2	0
Graphlet-67	0	0	0	0	2	0
Graphlet-68	12	12	12	0	0	0
Graphlet-80	1	1	1	0	0	0
Graphlet-82	0	0	0	0	1	0
Graphlet-85	0	0	0	0	2	0
Graphlet-86	0	0	0	0	2	0
Graphlet-125	0	0	0	0	0	1

**Table 4:** PN-GDDA results for comparison of structures A–F.

	A	B	C	D	E	F
Structure A	1000	0.877	0.864	0.797	0.802	0,887
Structure B		1000	0.954	0.840	0.745	0,782
Structure C			1000	0.880	0.742	0.768
Structure D				1000	0.782	0.819
Structure E					1000	0.853
Structure F						1000

**Figure 12:**
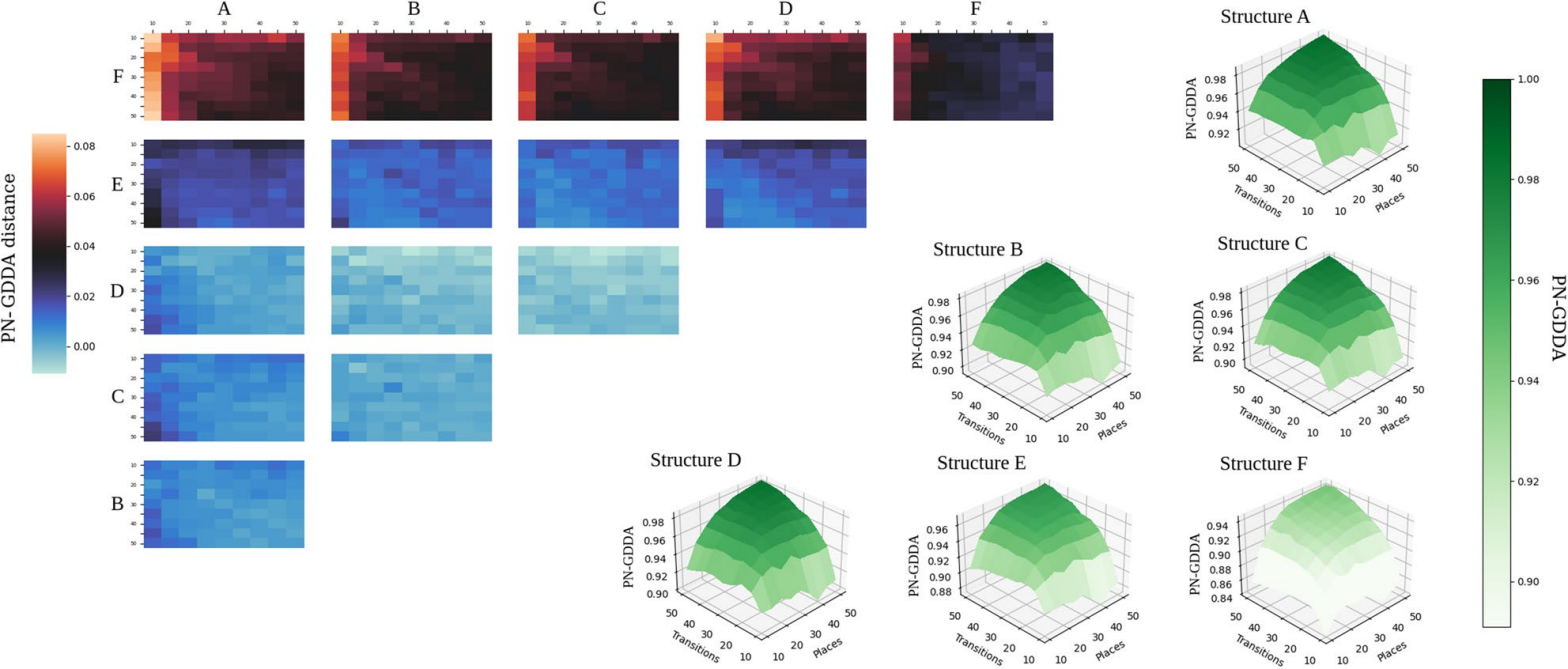
Diagrams in right-bottom part created for all extensions, show changes in behavior of GDDA in relation to net size (Data used for diagrams can be found in Tables S4–S9 from Supplementary Material). Heatmaps in right-top part show distance in GDDA values between each pair tested extensions^20^.

**Figure 13:**
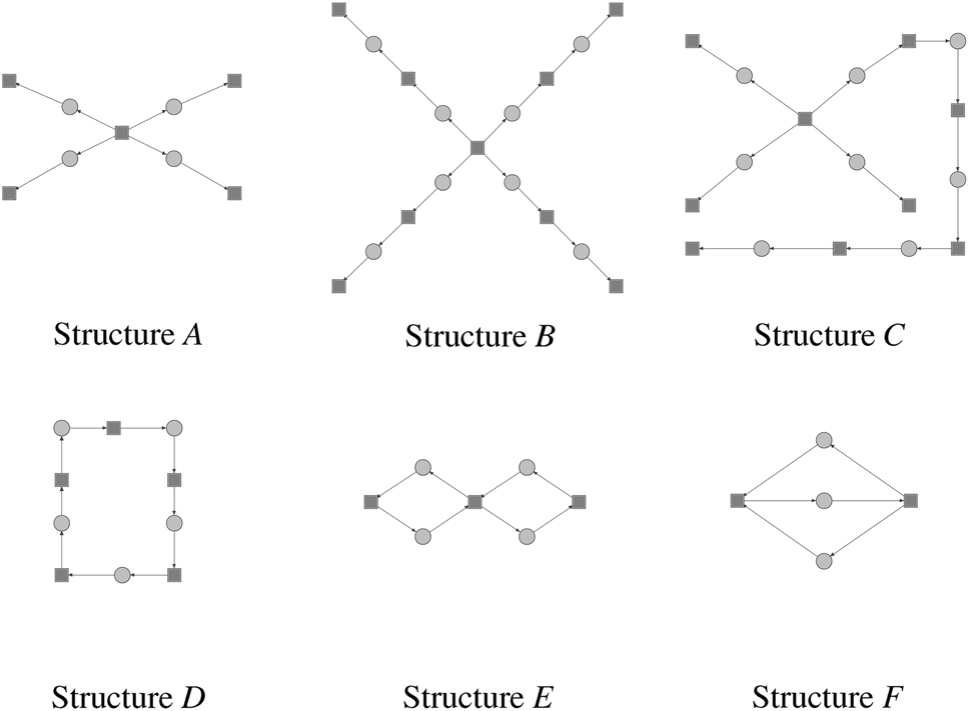
ADT-based structures used in the computational experiment.

To resolve this case, a next computational experiment was performed. In this experiment an impact of conADT-based differences on PN-GDDA was examined. For each random Petri net its duplicate extended by a specific ADT-based structure was generated. The conADT-based structures used in this experiment are shown in Fig. 13. Structures $A{-}E$ were connected by a single arc with the rest of the generated net. For case *F* we decided to connected the structure by 2 arcs and check its impact on PN-GDDA.

In Fig. 12 the results for each $A{-}F$ extension variant was presented in a separate diagram that represents the behavior of PN-GDDA value when the net size increases and the ratio of the numbers of places and transitions changes (the right bottom part of Fig. 12). To better present differences between each two variants a set of heatmaps was added (the left top part of Fig. 12).

An impact of structures $A{-}D$ on the PN-GDDA value is very similar, despite that they correspond to different graph structures (*D* is a cycle, $A{-}C$ are trees). It is a result of very similar sets of pn-graphlets that exists in those subnets. The largest level of differences through all test cases was observed between structure *F* and other extensions. It is caused by multiple connections to the original net. Each of those connections is responsible for new pn-graphlets that did not occur initially in the original net nor in its extension (Fig. 13).

**Figure 14:**
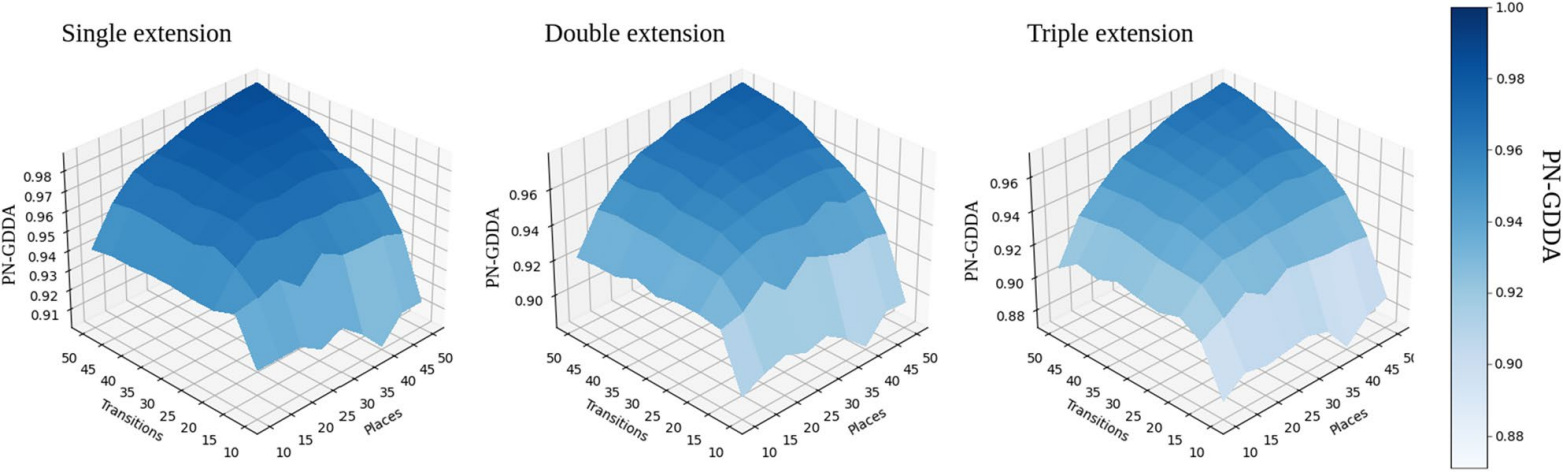
Changes of PN-GDDA value due to singular, multiple and triple extensions by the same *A* structure (Data used for diagrams can be found in Tables S4, S1, S11 from Supplementary Material)^20^.

To show a significance of multiple connection, a new test was performed. Each of the original test nets was extended multiple times by the same structure *A*—see Fig. 14. An impact on PN-GDDA of three *A* subnets connected by a single arc with the rest of the net was lower than for single structure *F* connected by two arcs. To properly describe that impact on PN-GDDA let us focus on the smallest case with 10 transitions and 10 places. Even when it was extended by 3 structures (resulting in 47-node net), its PN-GDDA value with relation to the base net (20-node net) was higher than the similarity between the base net and the one extended by structure *F* (25-node net).

The performed test showed that PN-GDDA is a measure which properly describes similarities between Petri net-based models of biological systems. It should be noted that such models have some characteristics (e.g., the ratio of the numbers of transitions and places) which models of other types (i.e., models of non-biological phenomena) may not have. The test also indicated that on the basis of PN-GDDA it is not possible to distinguish what is the structure of a difference between the compared Petri nets (however, it is also not possible in the case of the classical GDDA applied to PPI networks comparison).

All diagrams with the results from experiments, were generated using a Python data visualization library Seaborn (v0.11.2)^20^.

## Conclusions

The popularity of Petri nets creates a pressure for new and better analysis methods that could satisfy demands from newly explored areas of science. Unfortunately, Petri nets suffer from shortage of dedicated comparison algorithm and adaptation of graphlets can change this situation.

The process of adaptation to new environments and improvement of graphlets has been already started^15^ and in this paper we continue it by proposing a new variant of pn-graphlets dedicated for Petri nets. Moreover, we performed extensive tests to evaluate PN-GDDA metric, which resulted in confirmation of its potential and allowed to determine its limitations when used to compare Petri net-based models of biological systems. Additionally, the work on graphlets and its adaptation resulted in a new extension of our application Holmes, which is a platform for modelling, simulating an analysis of Petri nets^22^.

## Supplementary Information

Supplementary Information.

## Data Availability

The datasets generated and/or analysed during the current study are not publicly available due to their large size, but are available from the corresponding author on reasonable request.
