# Supplementary material for: Graphlets in comparison of Petri net-based models of biological systems
Source: Sci Rep. 2022 Dec 4;12:20942. doi: 10.1038/s41598-022-24535-5 (PMC9719902; doi:10.1038/s41598-022-24535-5)
Supplement: Supplementary file 1 — Supplementary Information. [file 41598_2022_24535_MOESM1_ESM.pdf]

Supplementary materials for article "Graphlets and Petri nets"

Table S 1: Data for Figure 9 - case for 3 node size graphlets

	10	15	20	25	30	35	40	45	50
10	0.701	0.793	0.774	0.802	0.77	0.756	0.777	0.755	0.786
15	0.774	0.799	0.829	0.812	0.831	0.81	0.806	0.791	0.829
20	0.793	0.755	0.833	0.85	0.849	0.857	0.842	0.862	0.849
25	0.758	0.82	0.832	0.84	0.868	0.846	0.859	0.86	0.843
30	0.755	0.818	0.83	0.86	0.885	0.857	0.877	0.872	0.887
35	0.773	0.801	0.825	0.779	0.883	0.88	0.891	0.895	0.881
40	0.731	0.74	0.804	0.838	0.865	0.896	0.881	0.89	0.879
45	0.684	0.785	0.808	0.864	0.871	0.861	0.886	0.895	0.894
50	0.67	0.777	0.82	0.829	0.872	0.886	0.878	0.885	0.906

Table S 2: Data for Figure 9 - case for 4 node size graphlets

p\t	10	15	20	25	30	35	40	45	50
10	0.597	0.674	0.689	0.671	0.657	0.628	0.669	0.665	0.687
15	0.693	0.698	0.728	0.692	0.685	0.693	0.676	0.689	0.734
20	0.721	0.704	0.73	0.754	0.74	0.742	0.732	0.725	0.756
25	0.707	0.731	0.751	0.765	0.771	0.768	0.747	0.733	0.738
30	0.712	0.755	0.756	0.789	0.794	0.755	0.775	0.787	0.794
35	0.704	0.753	0.758	0.737	0.812	0.797	0.827	0.821	0.798
40	0.701	0.728	0.734	0.774	0.794	0.826	0.816	0.811	0.818
45	0.651	0.743	0.761	0.806	0.802	0.757	0.812	0.819	0.836
50	0.65	0.73	0.771	0.799	0.825	0.827	0.81	0.815	0.829

Table S 3: Data for Figure 9 - case for 5 node size graphlets

p\t	10	15	20	25	30	35	40	45	50
10	0.569	0.64	0.629	0.611	0.616	0.577	0.603	0.582	0.62
15	0.645	0.668	0.671	0.67	0.656	0.649	0.614	0.62	0.655
20	0.68	0.675	0.686	0.708	0.676	0.684	0.645	0.67	0.668
25	0.684	0.691	0.703	0.723	0.736	0.707	0.701	0.683	0.689
30	0.66	0.673	0.699	0.72	0.709	0.699	0.701	0.725	0.734
35	0.648	0.689	0.718	0.685	0.727	0.743	0.733	0.752	0.743
40	0.671	0.682	0.697	0.701	0.73	0.744	0.752	0.741	0.748
45	0.616	0.686	0.707	0.726	0.731	0.692	0.757	0.764	0.741
50	0.624	0.654	0.7	0.708	0.743	0.747	0.752	0.77	0.784

Table S 4: Data for Figure 11 - case for A structure; Data for Figure 13 - case for single extension by A structure

p\t	10	15	20	25	30	35	40	45	50
10	0.925	0.925	0.925	0.918	0.923	0.914	0.902	0.914	0.914
15	0.947	0.949	0.953	0.944	0.955	0.949	0.948	0.948	0.944
20	0.943	0.958	0.966	0.963	0.965	0.963	0.964	0.961	0.96
25	0.943	0.963	0.97	0.974	0.972	0.974	0.974	0.971	0.967
30	0.944	0.959	0.97	0.974	0.976	0.976	0.979	0.976	0.97
35	0.944	0.96	0.968	0.975	0.978	0.98	0.979	0.981	0.979
40	0.943	0.961	0.97	0.976	0.979	0.982	0.982	0.98	0.982
45	0.943	0.961	0.968	0.975	0.978	0.982	0.983	0.985	0.984
50	0.941	0.959	0.97	0.974	0.978	0.982	0.984	0.986	0.987

Table S 5: Data for Figure 11 - case for B structure

p\t	10	15	20	25	30	35	40	45	50
10	0.913	0.917	0.917	0.911	0.911	0.904	0.893	0.901	0.905
15	0.936	0.939	0.947	0.939	0.948	0.941	0.941	0.937	0.936
20	0.937	0.951	0.959	0.957	0.957	0.957	0.958	0.955	0.954
25	0.935	0.952	0.963	0.972	0.967	0.968	0.966	0.964	0.962
30	0.932	0.953	0.964	0.969	0.973	0.971	0.975	0.972	0.966
35	0.929	0.952	0.962	0.969	0.972	0.977	0.977	0.978	0.975
40	0.93	0.953	0.963	0.97	0.975	0.977	0.978	0.978	0.979
45	0.926	0.951	0.962	0.97	0.972	0.98	0.979	0.981	0.982
50	0.927	0.947	0.959	0.969	0.974	0.978	0.982	0.981	0.983

Table S 6: Data for Figure 11 - case for C structure

p\t	10	15	20	25	30	35	40	45	50
10	0.911	0.913	0.915	0.91	0.911	0.903	0.891	0.902	0.902
15	0.933	0.943	0.947	0.936	0.946	0.94	0.941	0.937	0.935
20	0.934	0.948	0.959	0.957	0.957	0.956	0.959	0.952	0.953
25	0.932	0.95	0.961	0.965	0.966	0.966	0.966	0.963	0.962
30	0.929	0.949	0.96	0.969	0.97	0.968	0.972	0.971	0.965
35	0.928	0.949	0.962	0.969	0.972	0.975	0.976	0.978	0.975
40	0.93	0.949	0.961	0.969	0.973	0.976	0.977	0.977	0.977
45	0.923	0.948	0.961	0.967	0.973	0.977	0.979	0.981	0.981
50	0.919	0.943	0.958	0.967	0.973	0.978	0.979	0.981	0.983

Table S 7: Data for Figure 11 - case for D structure

p\t	10	15	20	25	30	35	40	45	50
10	0.92	0.919	0.924	0.918	0.922	0.913	0.899	0.909	0.911
15	0.936	0.948	0.954	0.945	0.955	0.946	0.945	0.943	0.944
20	0.94	0.951	0.961	0.962	0.962	0.96	0.961	0.957	0.956
25	0.933	0.954	0.964	0.97	0.971	0.972	0.971	0.968	0.967
30	0.93	0.951	0.963	0.97	0.973	0.975	0.976	0.974	0.97
35	0.933	0.953	0.962	0.971	0.977	0.977	0.977	0.978	0.977
40	0.932	0.951	0.962	0.97	0.975	0.977	0.98	0.98	0.981
45	0.927	0.948	0.961	0.968	0.973	0.979	0.981	0.982	0.983
50	0.923	0.948	0.96	0.97	0.974	0.979	0.981	0.983	0.984

Table S 8: Data for Figure 11 - case for E structure

p\t	10	15	20	25	30	35	40	45	50
10	0.9	0.898	0.899	0.894	0.897	0.885	0.873	0.884	0.887
15	0.921	0.926	0.932	0.926	0.935	0.929	0.923	0.925	0.922
20	0.925	0.939	0.946	0.944	0.946	0.944	0.942	0.941	0.941
25	0.92	0.942	0.951	0.954	0.952	0.955	0.953	0.95	0.95
30	0.919	0.94	0.953	0.956	0.958	0.958	0.959	0.958	0.952
35	0.916	0.942	0.952	0.958	0.962	0.962	0.961	0.967	0.961
40	0.915	0.943	0.953	0.959	0.962	0.965	0.963	0.963	0.963
45	0.911	0.94	0.953	0.96	0.962	0.964	0.967	0.968	0.969
50	0.905	0.938	0.95	0.961	0.965	0.967	0.968	0.968	0.97

Table S 9: Data for Figure 11 - case for F structure

p\t	10	15	20	25	30	35	40	45	50
10	0.84	0.862	0.867	0.863	0.865	0.856	0.845	0.851	0.86
15	0.866	0.881	0.9	0.894	0.904	0.897	0.898	0.898	0.897
20	0.871	0.89	0.905	0.913	0.917	0.916	0.917	0.916	0.914
25	0.871	0.9	0.912	0.918	0.921	0.926	0.928	0.928	0.923
30	0.867	0.904	0.917	0.923	0.926	0.928	0.933	0.934	0.93
35	0.866	0.904	0.916	0.925	0.928	0.932	0.936	0.939	0.938
40	0.863	0.904	0.923	0.928	0.929	0.934	0.936	0.937	0.941
45	0.861	0.904	0.918	0.932	0.933	0.936	0.94	0.943	0.945
50	0.857	0.9	0.919	0.932	0.939	0.941	0.943	0.943	0.944

Table S 10: Data for Figure 13 - case for double extension by A structure

p\t	10	15	20	25	30	35	40	45	50
10	0.894	0.9	0.904	0.899	0.902	0.894	0.883	0.897	0.896
15	0.925	0.928	0.939	0.928	0.94	0.935	0.931	0.933	0.93
20	0.927	0.941	0.947	0.951	0.949	0.946	0.953	0.947	0.948
25	0.927	0.942	0.953	0.956	0.959	0.96	0.961	0.96	0.956
30	0.924	0.942	0.954	0.96	0.963	0.963	0.966	0.965	0.962
35	0.928	0.944	0.954	0.96	0.967	0.969	0.968	0.969	0.97
40	0.923	0.944	0.958	0.964	0.966	0.97	0.971	0.971	0.974
45	0.923	0.945	0.955	0.963	0.965	0.971	0.974	0.976	0.976
50	0.922	0.943	0.955	0.964	0.967	0.972	0.973	0.976	0.978

Table S 11: Data for Figure 13 - case for triple extension by A structure

p\t	10	15	20	25	30	35	40	45	50
10	0.88	0.89	0.887	0.889	0.891	0.887	0.871	0.883	0.888
15	0.908	0.915	0.924	0.916	0.925	0.923	0.921	0.921	0.919
20	0.916	0.928	0.936	0.936	0.938	0.938	0.939	0.939	0.938
25	0.912	0.929	0.941	0.945	0.946	0.95	0.952	0.949	0.949
30	0.914	0.93	0.944	0.951	0.953	0.955	0.959	0.958	0.954
35	0.917	0.933	0.942	0.953	0.954	0.96	0.963	0.966	0.96
40	0.912	0.935	0.945	0.953	0.956	0.961	0.962	0.962	0.966
45	0.915	0.933	0.944	0.953	0.959	0.962	0.965	0.968	0.97
50	0.906	0.931	0.943	0.954	0.958	0.964	0.968	0.969	0.972
